# Community-based organizations’ perspectives on piloting health and social care integration in North Carolina

**DOI:** 10.1186/s12889-023-16722-4

**Published:** 2023-10-04

**Authors:** Raman Nohria, Junette Yu, Karissa Tu, Grace Feng, Shemecka Mcneil, Fred Johnson, Michelle Lyn, Karen Scherr

**Affiliations:** 1 Department of Family Medicine and Community Health, Duke University School of Medicine, 2100 Erwin Road, 27705 Durham, NC USA; 2 University of Washington School of Medicine, 1959 NE Pacific St, Seattle, WA 98195 USA; 3 University of California San Diego School of Medicine, 9500 Gilman Dr, La Jolla, 92093 CA San Diego, USA; 4 Slice 325, Durham, NC USA

**Keywords:** Social determinants of health, Health-related social needs, Medicaid population, Health and social care integration

## Abstract

**Background:**

Community-based organizations (CBOs) are key players in health and social care integration initiatives, yet little is known about CBO perspectives and experiences in these pilot programs. Understanding CBO perspectives is vital to identifying best practices for successful medical and social care integration.

**Methods:**

From February 2021 to March 2021, we conducted surveys with 12 CBOs that participated in the North Carolina COVID-19 Social Support Program, a pre-pilot for North Carolina’s Medicaid Sect. 1115 demonstration waiver program that addresses social drivers of health.

**Results:**

CBO participants preferred communication strategies that involved direct communication and felt clear communication was vital to the program’s success. Participants expressed varied experiences regarding their ability to handle a changing volume of referrals. Participants identified their organizations’ strengths as: strong organizational operations, past experiences with and understanding of the community, and coordination across organizations. Participants identified challenges as: difficulty communicating with clients, coping with capacity demands for scaling services, and lack of clear processes from external organizations. Almost all CBO participants expressed enthusiasm for participating in similar social care transformation programs in the future.

**Conclusions:**

CBO participants in our study had broadly positive experiences in the pilot program and almost all would participate in a similar program in the future. Participants provided perspectives that can inform health and social care integration initiatives, including strengths and challenges in such programs. To build and sustain health and social care integration programs, it is important to: (1) support CBOs through regular, direct communication that builds trust and power-sharing between CBO and health care entities; (2) leverage CBO community expertise; and (3) pursue an individualized assessment of CBO capacity and identify CBO capacity-building strategies that ensure program success and sustainability.

**Supplementary Information:**

The online version contains supplementary material available at 10.1186/s12889-023-16722-4.

## Background

There has been a strong push for health care organizations to integrate social care services in order to reduce costs and improve care quality [1, 2]. Early data suggests providing social care services (e.g., housing and nutrition) to Medicaid managed care members at-risk for homelessness or food insecurity results in reduced health care utilization [3–6] and net savings for Medicaid managed care models [4, 6, 7]. While health care leaders view integration with significant interest, health care leaders also recognize that buy-in and engagement from community-based organizations (CBOs) are needed for successful integration [8–12]. We define CBOs in this study as non-governmental, non-profit organizations overseen by an elected board of directors that partner with their local community to address community needs [13]. CBOs serve as the key entity for patients to receive the benefits of social care; these benefits include, but are not limited to: emergent social services, culturally relevant knowledge, and social support [14]. CBOs are also not uniform: due to chronic underfunding and limited staff, CBOs have varying capacities to provide services, incorporate funding streams, and receive referrals [8–10, 14, 15].

While the literature on CBO perspectives is emerging, [15–18] there has been minimal peer-reviewed literature on the impact of real-world implementation of health and social care integration on CBOs. Understanding CBO perspectives is vital to identifying best practices for successful health and social care integration. To address this gap, we elicited CBO perspectives on the implementation of the North Carolina COVID Support Services Program (SSP), a pre-pilot for North Carolina’s Medicaid Sect. 1115 demonstration waiver program [10].

## Methods

### Study context

This study focuses on the implementation of the Duke Health COVID SSP collaborative. Funded by the North Carolina Department of Human and Health Services (NCDHHS) through the Coronavirus Aid, Relief, and Economic Securities (CARES) Act of 2020, [19] the Duke Health COVID SSP collaborative was designed to support residents in quarantine or isolation due to COVID-19 [20]. This program served as a pre-pilot for the North Carolina Medicaid Healthy Opportunities Program, North Carolina’s Medicaid Sect. 1115 demonstration waiver program [10]. The Duke Health COVID SSP collaborative was comprised of Duke Health, NCDHHS, CBOs, and community-health workers (CHWs). Faculty in the Duke Health population health management office at a large academic health system served as the backbone organization for the collaborative. NCDHHS required identifying a backbone organization that would act as the primary collaborative driver for implementation of SSP. The backbone organization’s role was to: receive client referrals, screen referrals for program eligibility, send appropriate referrals to CBOs to deliver services, and oversee CBO reimbursement for service provision. CHWs were funded by a different contract.

The Duke Health COVID SSP was implemented from August 1, 2020 to March 31, 2021 for the following 7 North Carolina counties: Durham, Franklin, Granville, Nash, Vance, Wake, and Warren. The Duke Health COVID SSP partnered with 19 CBOs, 12 of which were led by historically underrepresented minorities, 8 were led by women, and most (16/19) had at least one English-Spanish bilingual staff (see Table 1 for organization characteristics). Examples of CBOs in our study include ministries, farmer cooperatives, and agencies that specialize in community development, food delivery, culinary education, and/or non-emergency medical transportation. CBOs were contracted to deliver social needs services, including food (i.e., food boxes and prepared meals), transportation, medication delivery, and COVID supply kits containing items such as face masks, thermometers, and hand sanitizer. Services could be extended beyond 14 days if additional time in quarantine was recommended by a health professional. Program referrals were received from either the large academic health system or the county health departments.

**Table 1:** Characteristics of participating CBOs

CBO Name	Services	Counties Served	Minority Led	Female Led	Bilingual/ Bicultural staff	Date Joined
**Responded to survey**						
Organization #1	Food box delivery, Covid-19 supplies	Granville, Vance		√	√	Sep 2020
Organization #2^a^	Food box delivery, Covid-19 supplies	Durham	√	√	√	Sep 2020
Organization #3	Food box delivery	Durham			√	Sep 2020
Organization #4	Food box delivery, Meal delivery, Covid-19 supplies	Durham, Wake	√		√	Sep 2020
Organization #5^a^	Food box delivery, Covid-19 supplies	Durham, Franklin, Granville, Vance	√	√	√	Sep 2020
Organization #6^a^	Food box delivery	Durham, Franklin, Granville, Nash, Vance, Wake, Warren	√		√	Sep 2020
Organization #7	Meal delivery	Durham			√	Sep 2020
Organization #8^a^	Food box delivery, Covid-19 supplies	Durham, Franklin, Granville, Nash, Vance, Wake, Warren	√	√	√	Sep 2020
Organization #9	Food box delivery, Covid-19 supplies	Durham, Granville, Warren	√			Sep 2020
Organization #10	Food box delivery, Covid-19 supplies	Durham, Wake	√		√	Nov 2020
Organization #11	Food box delivery	Durham, Granville, Vance, Wake	√		√	Nov 2020
Organization #12	Covid-19 supplies	Durham, Franklin, Granville, Nash, Vance, Wake, Warren			√	Jan 2021
**Did not respond to survey**						
Organization #13^a^	Food box delivery, Meal delivery, Covid-19 supplies	Durham, Franklin, Wake	√	√	√	Sep 2020
Organization #14^a^	Food box delivery	Durham	√	√	√	Sep 2020
Organization #15	Covid-19 supplies	Granville, Franklin, Vance, Warren		√	√	Sep 2020
Organization #16^a^	Food box delivery	Durham, Franklin, Granville, Nash, Vance, Wake, Warren	√		√	Sep 2020
Organization #17	Transportation, Medication delivery	Durham, Granville, Vance	√			Nov 2020
Organization #18	Meal delivery	Durham, Franklin, Granville, Nash, Vance, Wake, Warren			√	Nov 2020
Organization #19	Transportation, Medication delivery	Durham, Granville	√	√		Nov 2020

The table includes all CBOs that served as service vendors and does not include partner organizations that only provided CHWs

^a^CBOs marked with an asterisk also had CHWs on their team

### Study design

This study was part of a larger evaluation of the North Carolina COVID SSP [21]. We surveyed CBOs that were subcontracted for the program after program completion. Program staff worked with CBO participants to design the survey. The survey instrument was then tested by program staff prior to implementation. Program staff approached eligible CBOs for survey completion from February 2021 to March 2021. This study was considered exempt by the Duke Health University Institutional Review Board.

### Measures

#### Preferences for support

We assessed CBO participant perspectives on different formats of communication and support from the backbone organization. CBO participants could access support from the Duke Health team during SSP via direct communication (weekly town hall meetings, email, and phone/Zoom) and indirect communication (NCCARE360, program-specific website, and the Wayfinder chart infographic). The program-specific website and Wayfinder were created by the Duke Health team to provide CBO participants with guidance on how to create, process, and categorize NCCARE 360 referrals.

We assessed CBO participant perspectives on the helpfulness of each of these resources with the following question: “Rank your experience using these support resources or platforms during SSP:” with the answer choices *very helpful, somewhat helpful, not very helpful, and did not use/access this*. We asked CBO participants to provide additional feedback on their answers in an open-ended survey question.

We also assessed CBO participant’s overall perspective of support. We asked CBO representatives to respond to the following statement: “My previous relationship with the Duke Health Support Team made it easier to partner with SSP” with a Likert-scale response: (1) *strongly agree* (2) *somewhat agree* (3) *neither agree nor disagree* (4) *somewhat disagree or* (5) *completely disagree*. We also asked participants “What were some ways that the Duke Health team has been the most helpful to your organization? (Give specific examples).” Finally, we asked, “If there were a similar program in the future, how would you like the Duke Health team to support you more effectively? (Give specific examples).”

#### Changing referral volume

To assess CBO participant perspectives on changing referral volume, we asked “How did your organization feel about the unfixed volume of referrals sent to you on a daily/weekly basis?” They indicated whether they: (1) *Liked working with changing weekly/daily volumes* (2) *Were able to adapt, but preferred a steady, fixed volume; or* (3) *Struggled with the fluctuation of referrals and would need a fixed, steady volume for similar programs in the future*.

#### Strengths and challenges of SSP participation

To assess CBO participant perspectives on strengths and challenges, we asked: (1) “What were your organization’s major strengths in your partnership with SSP?”; (2) “What challenges did your organization face in your partnership with SSP?”; and (3) “What would your organization do differently knowing what you know now?”

#### Reflections on future participation

To understand CBO participant perspectives on future participation, we asked: (1) “If there were a similar program again, would you provide the same services? Why or why not?”

### Analysis

For qualitative responses, participant answers were copied into excel. KS and RN used an iterative inductive content analysis approach to categorize and identify themes within responses. KS developed the initial coding structure, which was reviewed and adjusted by RN. After application of the coding themes, the data was reviewed by team member JY to ensure they agreed with the findings. Final adjustments were made using an iterative process until all coders (KS, RN, and JY) agreed on the categorizations. Categories were not mutually exclusive and participants’ answers could address multiple themes. See Supplement 1 for all coded responses.

## Results

### Respondents

Of the 19 CBOs that participated in the program, 12 completed the survey. Table 1 describes the SSP role and organization characteristics for responders and non-responders. For the CBOs who responded to the survey, most (9/12) CBOs were minority led and nearly all (11/12) had bilingual/bicultural staff. Survey respondents’ roles were as follows: CEO or executive director (*n* = 7), Operations Lead (*n* = 2), and support staff (e.g. CHW coordinator, community support manager, and client services associate) (*n* = 3). To protect participants’ privacy, specific roles are not shared at the organization level.

### Preferences for support

Table 2 presents participants’ preferences for communication format from the backbone organization. Direct forms of communication (phone/zoom, email, and townhalls) were utilized by all participants and universally rated as very or somewhat helpful, whereas indirect forms of communication (NCCare360, website, and Wayfinder) were utilized by only some participants and rated more mixed in helpfulness. All participants (12/12) rated phone/Zoom as very helpful and most participants rated email and town halls as very helpful (11/12 and 7/12, respectively). One participant noted that “The weekly check-ins were crucial for disseminating important information, especially as circumstances changed so quickly with COVID…” Participants’ perceptions of indirect forms of communication were more mixed, with only some participants finding it helpful. For NCCARE360, most participants (9/12) found it at least somewhat helpful, but a few (2/12) rated it as “not very helpful” and one participant did not access it. One participant “really appreciated the NCCARE360 platform…[it] helped make things much easier for us as an agency.” In contrast, another participant noted that “unfortunately, nc360 was difficult for our team.” Fewer participants utilized the website and Wayfinder support tool; 8/12 participants never accessed the website and 4/12 never utilized Wayfinder. For those who did utilize these platforms, their perceived helpfulness was mixed.

**Table 2:** CBO’s perceived helpfulness of different communication formats

Communication Format	Very Helpful	Somewhat Helpful	Not Very Helpful	Did Not Use/Access
Phone/Zoom	12	0	0	0
Email	11	1	0	0
Town Halls	7	5	0	0
NCCARE 360	5	4	2	1
Wayfinder	5	2	1	4
Website	2	1	1	8

In terms of perspectives on overall support, 6/10 participants strongly agreed that their previous relationship with the Duke Health support team made it easier to partner with SSP, while 4/10 participants neither agreed nor disagreed (2 participants did not have a prior relationship with the team and did not answer the question). In terms of what participants in our study found most helpful from the backbone organization, nearly all participants (11/12) highlighted the helpfulness of regular communication and accessibility for questions. For example, one participant noted “Holding weekly/bi-weekly calls were great in terms of providing information and solidifying respectful relationships among the CBOs…” Participants also appreciated help with networking, logistics, funding, and knowledge. In terms of how the backbone team could better support their CBOs, most participants (8/12) could not identify anything that the team could improve. The remaining participants (4/12) requested d improved support around issues of capacity and logistical support, particularly early in the program.

### Perspectives on changing referral volume

Participants were mixed in their preferences for working with a fixed versus steady volume of referrals. Half of participants (6/12) liked working with a changing weekly/daily volume of referrals, one third of participants (4/12) were able to adapt to a changing volume but preferred a steady, fixed referral volume, and the remaining participants (2/12) reported struggling with the changes in referral volume and that they would need a fixed, steady volume of referrals for similar programs in the future.

### Strengths and challenges of SSP participation

Participants identified three organizational strengths in their partnership with SSP: (1) strong organizational operations; (2) past experiences with the target community; and (3) networking and coordinating with other organizations. Participants highlighted many organizational operations that were sources of strength, including communication (“communicating and addressing any concerns in a timely manner”), organization (“being able to multi-task”), adaptability (“capacity to quickly ramp up service), and clear work flow processes (“internal processes made it easy to respond quickly to questions”). Four participants noted that their past experiences in and knowledge of the target communities were a key strength. For example, one participant highlighted that their strength was “knowing the communities we serve [and] ability to adapt to the client’s needs (disabled, Spanish speaking only, navigating around clients having no telephones, bed ridden).” Finally, two participants noted that an important strength of the project was networking and coordinating stakeholders so that their “scope and reach expanded.”

Nearly all participants (11/12) identified at least one challenge they faced during the partnership. Participants identified three challenges in their partnership with SSP: (1) communication with clients; (2) coping with capacity demands; and (3) lack of clear processes/guidance from external organizations. Five participants raised issues of communicating with clients, including issues with language barriers, missing or outdated client contact information, or clients not answering phone calls. Five participants noted issues related to coping with capacity demands, such as difficulties with staffing (“we needed more hands”), acquiring inventory (“acquiring dry goods… trying to purchase them from stores was difficult”), adjusting to referral volume (“adjusting to the large volume of referrals sent to our agency each day/week to make sure each and every person/household was served.”), and invoicing (“lack of coordinated system for deliveries that linked to invoicing”). One participant summarized their issues with coping with capacity as “ like learning to swim by being thrown in the deep end of the pool.” Finally, four participants noted challenges with a lack of clear processes/guidance from external organizations, which included issues in communication with the Duke Health support team, and the “state’s lack of clarity and shifting eligibility on [CARES Act] relief.”

Reflecting on their experiences, participants would change three main processes: (1) hire more staff, (2) improve their management of referral volume and inventory; and (3) invest in better documentation and tracking systems. Two participants recognized the importance of hiring more staff, including bilingual staff, CHWs, volunteers, and drivers. Six participants expressed a desire to improve their management of referrals or inventory, with several noting a need to better recognize their limits in terms of referral volume. For example, one noted they would “reasonably regulate supply/production rather than try to meet demand at all costs.” Four participants discussed a need for improved documentation systems, such as a need to “establish forms to be shared among all workers from the beginning,” “create a tighter workflow process to track attestation forms and referrals,” and “use a different monitoring system [to avoid] duplicated services…and catch errors.”

### Reflections on future participation

If offered the opportunity to participate in a similar program, all but one participant expressed enthusiasm. Participants recognize that there is significant unmet social need in the community and programs like SSP highlight the value CBOs in our study provide to the community. One participant stated “We enjoy what we do and will help in whatever way possible to assist those in need.” Participants also enjoyed the opportunity to collaborate with other program entities and the advantage such partnerships provided to address needs at-scale. Two participants noted that although they would participate again, they would want to shift their role to coordination or health education in future programs. The one participant that did not have interest in participating in a future program noted: “Since we are in a phase where Covid 19 efforts are changing, we are planning on keep serving our community in other ways than delivering food.”

## Discussion

This study examined CBO perspectives on real-world implementation of a health and social care integration program. Our study adds to the emerging literature on CBO and health care partnerships for health and social care integration [15–18]. CBO participants in our study had broadly positive experiences in the pilot program and almost all would participate in a similar program in the future. Participants preferred communication strategies that involved direct communication. Participants viewed changes in referral volume differently and those participants that struggled highlighted their limitations in capacity as the main barrier to scaling services. Participants also provided strengths and challenges that can inform health and social care integration initiatives. We expand upon our findings below:

### Participants would engage in future health and social care integration programs

Participants in our study, like CBOs in other studies, [15, 18] demonstrated enthusiasm for participating in similar social care transformation programs in the future. CBO participants in our study feel that these programs provide an opportunity to demonstrate CBO value to non-CBO entities and allow CBOs to partner and address needs at a scale larger than an individual CBO.

### Participants value direct communication

We found that CBOs preferred communication strategies that involved direct communication. While we cannot directly infer why communication was critical for our participants, other studies on communication in health and social care integration reveal that communication is a critical component in brokering trust in these nascent partnerships [16, 22–26] and is particularly valued by CBOs as a means to encourage power-sharing [15, 24, 27]. We hypothesize that communication is considered highly effective in health and social care partnerships if it is bi-directional, transparent, and continuous [28]. Although some participants found the non-direct forms of communication and support helpful, participants noted that these forms of support were less frequently accessed compared to direct communication.

The Collective Impact Framework (CIF), [29] a framework that we used in our pilot, has been used in other social care transformation programs to promote communication between stakeholders through a backbone organization [30–32] and requires strong financial support, clear strategic vision, and engaged community stakeholders to maintain sustainability [33]. The Duke Health SSP program used a health system subsidiary as the backbone organization; other pilots have utilized a neutral convener [32]. Additional research is needed to identify the optimal choice for a backbone organization [31]. Other frameworks, such as those developed by the BUILD Health Challenge, [27, 34] Robert Wood Johnson Foundation’s Aligning Systems for Health, [35] Data Across Sectors for Health, [36] and HealthBegins, [37] could also provide value for understanding how to measure direct communication and the impact of communication on CBO and health care partnership success. Future research can assist with identifying strategies that promote direct communication in these partnerships and its correlation with partnership equity.

### Participants identify their past experiences working with and understanding of the community as a core strength

Participants in our study felt that their expertise in the community was a significant asset that they brought to a health and social care integration program. This finding is consistent with other studies on CBO expertise in health and social care programs [14, 15, 23]. CBO expertise in these studies includes: community mobilization, [23] resource identification, [14, 15] social support, [14] and resource and community navigation [14]. One potential explanation for why CBOs are particularly valuable to health care efforts is that CBOs reach the most marginalized and vulnerable populations and can elevate the role of the community in reducing health disparities [13]. Although CBOs do have community expertise, we suspect that CBO capabilities remain poorly understood by health care organizations [16, 17]. It is important for CBO and health care partnerships to leverage this CBO strength, which may be accomplished by fostering trust and building partnership equity [15].

### Participants had mixed opinions regarding referral volume changes and identified coping with capacity demands as a core challenge

As social needs referrals are scaled, health and social care integration programs will depend upon CBOs to meet this referral demand and cope with a changing volume of referrals. We found that participants viewed changes in referral volume differently, with some liking the changing referral volume while others preferred or even needed a steady, fixed volume of referrals. Participants that struggled to cope with a changing volume of referrals highlighted their limitations in capacity as the main barrier to scaling services. Study participants identified that they would hire more staff and tell the health care partner up-front their capacity for referrals. Other studies on health and social care integration have found similar results: [15, 16] while CBOs would like to partner in health and social care programs, CBO capacity may not match CBO capability [16].

To sustain health and social care programs, we propose that CBO and health care partnerships should conduct an early, individualized assessment of CBO capacity as part of program design and implementation. The Getting to Outcomes framework highlights 7 CBO capacity domains that can be assessed as part of program implementation [38]. Participants in our study identified the need for adequate staff numbers and technical resources as important domains to assess with capacity. Future research should focus on identifying the capacity domains most closely linked to health and social care integration program sustainability and effectiveness.

In addition to assessing CBO capacity, CBO and health care partnerships may benefit from understanding how to build CBO capacity. A study of the National Cancer Prevention and Control Research Network’s effort to implement evidence-based interventions in community settings offers five CBO capacity-building strategies [39]. With respect to training and communication strategies for capacity-building, participants in our study identified direct communication strategies, such as weekly town halls and phone/Zoom, as particularly helpful.

We also hypothesize that funding is an important capacity-building strategy due to the anticipated rise of social needs referrals [15–18, 40]. The lack of funding for CBO capacity in health and social care integration programs may be exacerbated by the financial incentives for health care organizations. Health care organizations might view program funds as a means to achieve organizational budgeting needs and hesitate to partner with CBOs without a clear return on investment that benefits the health care organization [17].

## Limitations

Our study has several limitations. First, our study did not include direct interaction with participants and thus we were not able to probe for more specifics but were rather limited by whatever information participants included in their survey responses. Future research should utilize qualitative methods such as focus groups and key informant interviews to gain additional insight, such as what aspects of direct communication can be utilized for future pilots and how subsidizing CBO capacity affects program outcomes. Second, our surveys were conducted with one group of stakeholders that served one region, which may limit its generalizability to other regions. Third, of the 19 CBO participants in our program, only 12 CBOs participated in the survey. Our study may not have captured the full breadth of CBO perspectives on our program’s implementation. Due to limited variability in CBO characteristics and small sample size, we were not able to assess for differences in CBO experiences and preferences as a function of their characteristics. Future research could focus on this. Fourth, our survey participants primarily served in leadership roles and their responses may not capture the perspectives of support staff who implemented the program. Fifth, the term “changing referral volume” was not clearly defined and may refer to either positive or negative fluctuations.

## Conclusions

While interest in health and social care integration programs is rapidly growing, such programs must acknowledge the perspective and role CBOs play in ensuring that these programs succeed. Our study examined a real-world implementation of a health and social care program through the lens of CBO participants. CBO participants in our study had broadly positive experiences in the pilot program and almost all would participate in a similar program in the future because they recognize significant unmet need in the community and the value that these programs can provide to the community. Participants highlighted the importance of direct communication to program implementation and provided perspectives on implementation strengths and challenges that can inform future health and social care integration initiatives. To build and sustain health and social care integration programs, it is important to: (1) support CBOs through regular, direct communication that builds trust and power-sharing between CBO and health care entities; (2) leverage CBO community expertise; and (3) pursue an individualized assessment of CBO capacity and identify CBO capacity-building strategies that ensure program success and sustainability. Future work should examine policy frameworks that identify strategies to support CBO capacity and encourage direct communication between CBOs and health care organizations.

### Supplementary Information

**Additional file 1. **Coded qualitative responses. 

## Data Availability

All data generated or analyzed during this study are included in this published article and its supplementary information files. For any requests about the data from this study, please contact Raman Nohria at raman.nohria@duke.edu.

## Abbreviations

CBOs: Community-based Organizations
SSP: Support Services Program
CARES: Coronavirus Aid, Relief, and Economic Securities
CHWs: Community-health Workers
CIF: Collective Impact Framework
