# Supplementary material for: Community-based organizations’ perspectives on piloting health and social care integration in North Carolina
Source: BMC Public Health. 2023 Oct 4;23:1914. doi: 10.1186/s12889-023-16722-4 (PMC10548645; doi:10.1186/s12889-023-16722-4)
Supplement: Supplementary file 1 — Additional file 1. Coded qualitative responses. [file 12889_2023_16722_MOESM1_ESM.docx]

**Supplement 1. Coded qualitative responses.**

**“What were some ways that the [Blinded] team has been the most helpful to your organization?**

1. All responses mentioned communication as a helpful form of support.
   1. Communication to provide logistical assistance
      1. The REDACTED has been amazing! Detail-oriented, communicative, and supportive. They helped us connect with other organizations to get rid of surplus food boxes, assigned referrals when we needed to get rid of surplus produce, and partnered with us on the COVID testing site distribution. They went above and beyond with the weekly town hall meetings and seamless invoicing and financing.
      2. REDACTED has been helpful in sending over names for referrals and locating letters of authorization for SSP clients. REDACTED has been helpful in navigating the REDACTED system, budgeting, and answering questions via email.
      3. REDACTED was very helpful, REDACTED was AWESOME, REDACTED helped me navigate through spreadsheets and other relative information
   2. Communication to share knowledge and information
      1. REDACTED addressed our concerns and answered any questions we had as it related to the partnership
      2. The REDACTED was most helpful by keeping an open line of communication that was clear. We had a few tough conversations but overall they were easy to work with and are very knowledgable.
   3. Having an open, positive attitude in communications
      1. After January, REDACTED began overcommunicating which was helpful for a new and growing program. Since the beginning, REDACTED always made themselves available to jump on a chat if things need to be discussed. They made themselves available and had positive attitudes even when things may have been a bit bumpy.
      2. REDACTED was most helpful by keeping an open line of communication that was clear. We had a few tough conversations but overall they were easy to work with and are very knowledgable.
      3. REDACTED remained accessible throughout the project and their follow up to any concerns assisted us in being able to provide services for clients especially those that were difficult to make initial contact with
   4. Communication to facilitate networking with other organizations
      1. Accessible, patient, knowledgeable, friendly. REDACTED helped us with reimbursement requests and getting set up on Google Box. REDACTED brought the Y into the project and connected us with other CBOs.
      2. Increased exposure in the community and beyond. Provided some funding that will allow more people to be served as a result of REDACTED’s participating in the program. Introduction to other community-based organizations. Direct opportunity to impact social determinants of health
      3. Holding weekly/bi-weekly calls were great in terms of providing information and solidifying respectful relationships among the CBOs
   5. Communication (No further specifications)
      1. Communication
      2. Great communication!!!
2. Funding
   1. Increased exposure in the community and beyond. Provided some funding that will allow more people to be served as a result of REDACTED’s participating in the program. Introduction to other community-based organizations. Direct opportunity to impact social determinants of health

**“If there were a similar program in the future, how would you like the [Blinded] team to support you more effectively? (Give specific examples).”**

1. Earlier logistics planning
   1. I think it may be helpful to find 1 to 3 organizations who can help provide initial and ongoing feedback, and see how that feedback can be incorporated into immediate changes to help improve the overall process and workflow.
   2. It appears any challenges we encountered were a product of entering the program when it was more the half completed and numbers were very high. Participation from the beginning would have addressed our challenges.
   3. Establish more quality control checks and balances earlier in the program.
2. Capacity management
   1. We are thankful for all the support from REDACTED, just keep the good communication. Maybe if the coverage area is sectorized , would be easier to serve better our community with resources we have. We have presence in 3 counties and sometimes we struggle with the coverage
   2. Agree upon a set number of boxes or service capacity that we can handle ahead of time
3. Continued strong support
   1. REDACTED was accessible, efficient, and responsive. I would hope that these qualities would remain in a similar, future program.
   2. Effective and consistent communication always is helpful for partnerships to work well together.
   3. REDACTED would like continued support, REDACTED has been nothing but a blessing to our organization and we feel confident that we could continue a viable partnership
   4. REDACTED did a great job, keep that same effort and include us even more.
   5. We are thankful for all the support from REDACTED, just keep the good communication. Maybe if the coverage area is sectorized , would be easier to serve better our community with resources we have. We have presence in 3 counties and sometimes we struggle with the coverage
   6. REDACTED would prefer to continue to work the with REDACTED as with this project as a good working relationship has been developed We appreciate this opportunity and look forward to future collaborations with REDACTED
4. Unsure
   1. not sure we can chat
5. Nothing to change
   1. I would not change anything

**“What were your organization’s major strengths in your partnership with SSP?”**

1. Existing capacity (structure, processes, knowledge, connections) in the area, aka “strong organizational operations”
   1. being able to Multi task.
   2. the standardized process through which we order meal boxes from our caterer, ServTracker software
   3. We were also very good at demonstrating structure and financial management and accuracy.
   4. Capacity to quickly ramp up service. Back office support and production staffing.
   5. Internal processes made it easy to respond quickly to questions. This happened several times over the contract period
   6. Appropriate knowledge of emergency food box delivery Clear understanding of food cost and supplies needed for this task Clear understanding of national average cost per serving for food Proper storage and inventory for food
   7. Adequate ongoing volunteer support to meet the demands associated with clients needs
   8. We were good at communicating and addressing any concerns in a timely manner
   9. Acquiring produce from small- to mid-size NC farmers - Acquiring dry goods in partnership with local co-ops (although this was also a challenge; see below) - Packing food boxes (although we could have used more help) - Distributing food boxes (mainly at testing sites; at-home deliveries were a challenge)
   10. Ability to grow/adapt (subtheme)
       1. We are a dedicated team, we could figure out many logistics we didn't have before, we could serve many people we couldn't serve before, thanks to REDACTED, we discovered ways to work during pandemic times.
       2. ability to adapt to the client's needs (disabled, Spanish speaking only, navigating around clients having no telephones, bed ridden) and provided wrap around services.
2. Past experiences with the community and understanding their needs
   1. Knowing the communities we serve, ability to adapt to the client's needs (disabled, Spanish speaking only, navigating around clients having no telephones, bed ridden)
   2. Food inventory, 35 year history in the community specific territory covered--Granville County experience with pre-established portions for individuals and families cooperative CHWs and their engagement CHW Coordinator's leadership and involvement with REDACTED and REDACTED
   3. our ability to serve clients who may not be well enough to prepare meals for themselves by providing frozen meals rather than grocery boxes.
   4. The REDACTED’s reputation
3. SSP facilitated successful connections/coordination across organizations
   1. The ability to dialogue weekly to meet the needs of five different counties. The scope and reach expanded.
   2. Our major strength within this project was bringing other stakeholders to the table. With the opportunity from REDACTED, REDACTED was able to introduce 6-8 organizations to this opportunity which in turn kept their businesses afloat, employees paid and expanded services that could be provided to the community. We were able to implement an efficient food consortium that created jobs (packagers, delivery drivers, chefs, admin), Service provider organizations that were able to hire Community Health Workers and generate income.

**“What challenges did your organization face in your partnership with SSP?”**

1. Difficulty communicating with clients from 5 CBOs. Reasons included language barriers, contact info not updated, clients not answering phone.
   1. Some Language barriers with clients
   2. Communicating with clients, primarily after deliveries; communicating with them before took a lot of time but was manageable - Communicating with Spanish-speaking clients
   3. the volume of Spanish-speaking clients (we only have one Spanish-speaking staff member
   4. We also had a huge challenge truing contact the clients, some of them never answer the phone.
   5. There were a few instances where clients were difficult to reach via contact information listed. DSSP remained helpful in working with CHWs to vain access to updated client information
2. Need for new processes/structures/capacity within organization
   1. Lack of coordinated system for deliveries that linked to invoicing
   2. limited staff capacity for enrollment and delivery
   3. packing food boxes - we needed more hands
   4. Acquiring dry goods; eventually partnered with local co-op to buy, but trying to purchase them from stores was difficult)
   5. Before Covid 19, we did not deliver food. It was challenging with the storage of food, initially finding suppliers, finding storage and we even bought a van to be able to use on deliveries.
   6. Adjusting to the large volume of referrals sent to our agency each day/week to make sure each and every person/household was served
   7. Getting acclimated. It was like learning to swim by being thrown in the deed end of the pool.
3. Lack of clear processes outside organization (SSP support team and beyond)
   1. State's lack of clarity and shifting eligibility on relief payments during first semester
   2. We had major trouble accessing REDACTED which made it difficult for us to see what other CBO's were able to navigate.
   3. Another challenge was having a clear understanding who was the appropriate point of contact with SSP. As new team members joined, we did not know if they were the point person or providing support.
   4. Contract ending points were difficult
4. Other
   1. clients' limited freezer/refrigerator space

**What would your organization do differently knowing what you know now?”**

1. Increase staff
   1. Hire more bilingual staff
   2. Engage volunteers to help pack and distribute at testing sites - Find a creative way to conduct at-home deliveries (partner with transportation org, partner with CHWs, find funds to hire new driver, engage volunteer drivers, etc)
2. Improved management of referral volume
   1. Knowing what we know now, REDACTED would request a steadier stream of referrals so that we can allocate staff time appropriately.
   2. When the project started, the delivery times went to far late, our were exhausted .We would plan better the amount of deliveries.
   3. Reasonably regulate supply/production rather than try to meet demand at all costs.
3. Improve processes/structures, particularly early in process
   1. Inventory-related
      1. Have a back-up organization secured each week to take any left-over boxes
      2. Now that we know the volume level of potential referrals that could be sent, we know how often to restock our supplies to accommodate the number of referrals
      3. We would prepare a PPE inventory to meet the demand
   2. Documentation-related
      1. We would use a different monitoring system. For example we duplicated services on three separate occasions out of over 335 deliveries. We would like to have a better system to navigate and catch errors
      2. We would be more hands on with understanding and documenting paperwork with our CHW partners.
      3. We would also work more closely with SSP to create a tighter workflow process to track attestation forms and referrals
      4. Establish forms to be shared among all workers from the beginning
   3. Overall/better coordination
      1. I think it would be best to have a few organizations specializing in acquiring food products, another in delivering and distributing, and another in communicating with clients and conducting surveys. Having 100 organizations taking on each of these tasks is overwhelming, does not allow for quality control, and is unsustainable. Instead, we could have 10 procurement, 10 distribution, and 10 client communication organizations working together in respective geographic areas. This would allow each organization to use their time and resources more efficiently and ultimately impact more people. -> this answer is from the question about providing similar services in the future but I think it fits really well here, wondering if we can address that in the methods so we can use it here.
      2. Engage volunteers to help pack and distribute at testing sites - Find a creative way to conduct at-home deliveries (partner with transportation org, partner with CHWs, find funds to hire new driver, engage volunteer drivers, etc)

**“If there were a similar program again, would you provide the same services? Why or why not?”**

1. No – one organization that wants to shift focus
   1. Since we are in a phase where Covid 19 efforts are changing , we are planning on keep serving our community in other ways than delivering food
2. Yes – remainder
   1. Shift role: 2 organizations
      1. we would like to teach more than just give out
      2. I think it would be best to have a few organizations specializing in acquiring food products, another in delivering and distributing, and another in communicating with clients and conducting surveys. Having 100 organizations taking on each of these tasks is overwhelming, does not allow for quality control, and is unsustainable. Instead, we could have 10 procurement, 10 distribution, and 10 client communication organizations working together in respective geographic areas. This would allow each organization to use their time and resources more efficiently and ultimately impact more people. REDACTED would be able to provide local produce procurement (fruits, vegetables, milk, eggs, bread, staples) and deliveries. We would most likely need to hire a few more people, though, depending on the workload.
   2. Provide the same service – remainder or CBOs. Reasons fell into three categories (organizations’ responses could reference more than one category)
      1. Need/serve community
         1. Nutrition Services (healthy meals & groceries) remain a critical need in our communities…Our belief that food is medicine, defines our desire to continue these services to provide healthy foods to our clients.
         2. Will help in whatever way possible to assist those in need.
         3. Absolutely yes there is a great need for services in our community where there are many disadvantaged individuals
         4. Yes, because we believe there was a need to serve our community.
         5. we want to serve
      2. Have capacity/capability
         1. REDACTED has established itself as a reliable source for these services, and will be able to expand our services to positively impact even more people in need.
         2. this work easily fit into our facility and staffing structure.
         3. REDACTED has the capacity to participate again.
         4. We developed a work flow and an amazing team!
         5. REDACTED is very familiar with emergency food delivery and possess the skills necessary to deliver food safely and in a timely manner
      3. Enjoy work, particularly collaboration
         1. we enjoy working with our community and creating unique partnerships that offer opportunities for others.
         2. We enjoy what we do… Collaboration opportunities are always welcome and encouraged.
      4. Specific reason not stated (n=1)
         1. We would be interested in providing meal deliveries services again in a similar program.
